# Bacterial membrane activity of α-peptide/β-peptoid chimeras: Influence of amino acid composition and chain length on the activity against different bacterial strains

**DOI:** 10.1186/1471-2180-11-144

**Published:** 2011-06-22

**Authors:** Line Hein-Kristensen, Kolja M Knapp, Henrik Franzyk, Lone Gram

**Affiliations:** 1 Division of Industrial Food Research, National Food Institute, Technical University of Denmark, Søltofts Plads, bldg.221, 2800 Kgs. Lyngby, DK-Denmark; 2 Department of Medicinal Chemistry, Faculty of Pharmaceutical Science, University of Copenhagen, Universitetsparken 2, 2100 København Ø, DK-Denmark

## Abstract

**Background:**

Characterization and use of antimicrobial peptides (AMPs) requires that their mode of action is determined. The interaction of membrane-active peptides with their target is often established using model membranes, however, the actual permeabilization of live bacterial cells and subsequent killing is usually not tested. In this report, six α-peptide/β-peptoid chimeras were examined for the effect of amino acid/peptoid substitutions and chain length on the membrane perturbation and subsequent killing of food-borne and clinical bacterial isolates.

**Results:**

All six AMP analogues inhibited growth of twelve food-borne and clinical bacterial strains including Extended Spectrum Beta-Lactamase-producing *Escherichia coli*. In general, the Minimum Inhibitory Concentrations (MIC) against Gram-positive and -negative bacteria were similar, ranging from 1 to 5 μM. The type of cationic amino acid only had a minor effect on MIC values, whereas chain length had a profound influence on activity. All chimeras were less active against *Serratia marcescens *(MICs above 46 μM). The chimeras were bactericidal and induced leakage of ATP from *Staphylococcus aureus *and *S. marcescens *with similar time of onset and reduction in the number of viable cells. EDTA pre-treatment of *S. marcescens *and *E. coli *followed by treatment with chimeras resulted in pronounced killing indicating that disintegration of the Gram-negative outer membrane eliminated innate differences in susceptibility. Chimera chain length did not influence the degree of ATP leakage, but the amount of intracellular ATP remaining in the cell after treatment was influenced by chimera length with the longest analogue causing complete depletion of intracellular ATP. Hence some chimeras caused a complete disruption of the membrane, and this was parallel by the largest reduction in number of viable bacteria.

**Conclusion:**

We found that chain length but not type of cationic amino acid influenced the antibacterial activity of a series of synthetic α-peptide/β-peptoid chimeras. The synthetic chimeras exert their killing effect by permeabilization of the bacterial cell envelope, and the outer membrane may act as a barrier in Gram-negative bacteria. The tolerance of *S. marcescens *to chimeras may be due to differences in the composition of the lipopolysaccharide layer also responsible for its resistance to polymyxin B.

## Background

Antimicrobial peptides (AMPs) are host defence molecules that constitute an essential part of the innate immune system among all classes of life [1]. Most AMPs permit the host to resist bacterial infections by direct killing of invading bacteria or other microorganisms, however, many AMPs are also immuno-modulatory and thus enhance the host defence against pathogens [2-5].

In addition to their natural role in combating infections, AMPs are recognized as promising alternatives to conventional antibiotics for which development of resistance has become an ever-increasing concern [6-8]. Peptide based drugs are often hampered by a rapid *in vivo *degradation, however, this may be circumvented by stabilizing natural AMPs by single-site substitutions or by designing novel synthetic analogues with an altered backbone that confers complete stability to the compounds. Careful investigation of structure-activity relationships may eventually allow design of optimised antimicrobial compounds with high activity and minimal side effects [9-15].

Many AMPs fold into an amphipathic structure, and it is believed that this topology enables pore formation or disintegration of bacterial cell membranes leading to bacterial cell death. The amphipathic properties usually include cationic patches that promote interaction with the anionic bacterial membrane as well as hydrophobic patches that favor integration into the membrane. Since this is the most common mode of action for AMPs there has been an intense focus on their ability to adapt an amphipathic conformation [16,17]. In particular, design of peptides with a high propensity to fold into a helical amphipathic conformation has attracted considerable interest [13,18-20].

**Figure 1:**
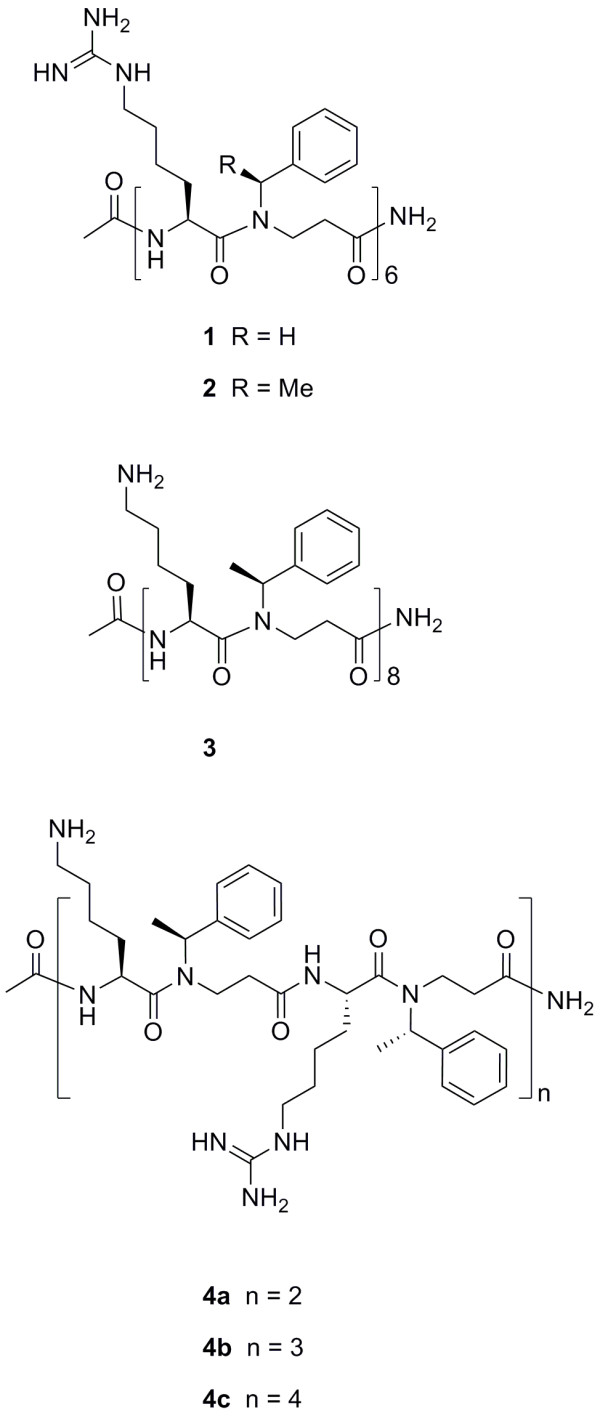
Chemical structure of the six α-peptide/β-peptoid chimeras

We have previously described a synthetic approach for design of α-peptide/β-peptoid chimeras possessing a design with alternating N-alkylated β-alanine (β-peptoid) and α-amino acid units (Figure 1). In addition, preliminary investigations showed that such peptidomimetics constitute a novel subclass of proteolytically stable antimicrobial compounds [21-23]. This design displays chiral unnatural β-peptoid residues that appear to contribute with structure-promoting effects and lipophilicity, while strongly cationic properties and intramolecular hydrogen bonding capacity are introduced via the α-amino acids lysine and/or homoarginine [24]. The precise secondary structure of these chimeras still remains to be elucidated, nevertheless, circular dichroism (CD) spectroscopy clearly indicates the presence of some degree of secondary structure [22,23]. Interestingly, a higher degree of secondary structure was found for analogues containing chiral side chains in the β-peptoid units (i.e. compounds 2 and 3 in Figure 1) as compared to chimeras with achiral β-peptoid residues (i.e. compound 1 in Figure 1) [22], but the effect of this on antibacterial activity remains largely unresolved [23].

The membrane-destabilizing effects of the chimeras have only been investigated in model liposomes prepared from phosphatidylcholine, a phospholipid found predominantly in eukaryotic cells, and several of the chimeras permeabilized such liposomal membranes [24]. Most studies on membrane activity of antimicrobial peptides have in fact been performed on model membranes [25-28] while the effects on cell membranes of viable bacteria have often not been tested. Also, the effect of membrane permeabilization on killing of bacteria has not been tested [27].

Here, we test the antibacterial effect of six chimeras against a spectrum of bacterial strains that include several important clinical and food-borne pathogens. The main purpose was to examine how the type of cationic amino acid and sequence length affected the antibacterial activity and to correlate this to a potential membrane-related mode of action in viable bacteria.

Part of this work was presented at the 50^th ^InterScience Conference on Antimicrobial Agents and Chemotherapy in Boston 12-15^th ^of September 2010.

## Methods

### Bacterial strains and culture conditions

Initial activity experiments were carried out with twelve strains from seven bacterial species representing common laboratory strains and clinical strains derived from both food-borne and nosocomial infections (Table 1). Stock cultures were stored at -80°C in 4% (w/v) glycerol, 0.5% (w/v) glucose, 2% (w/v) skimmed milk powder and 3% (w/v) tryptone soy powder. All experiments were carried out with bacteria incubated for one night (i.e. approximately 18 hours) at 37°C. Experiments were performed in cation-adjusted Mueller Hinton II broth (MHB) (Becton Dickinson 212322) adjusted to pH 7.4 or Tryptone Soy Broth (TSB) (Oxoid CM0129) for the ATP leakage assays. Brain Heart Infusion (BHI) (CM1135) with agar (VWR 20768.292) 1.5% as gelling agent was used throughout for colony plating.

**Table 1:** Origin and reference of bacterial strains used in the present study

	Origin	Ref
*S. aureus *8325-4	Wildtype	[59]
*K. pneumoniae *ATCC 13883	Human, clinical	-
*S. marcescens *ATCC 8100	Human, clinical	-
*E. coli *ATCC 25922	Wildtype	-
*E. coli *MG1655	K-12 F^- ^lambda^-^	[60]
*E. coli *AAS-EC-009	Human, clinical	^a^
*E.coli *AAS-EC-010	Human, clinical	^a^
*L. monocytogenes *4446	Human, clinical	[61]
*L. monocytogenes *N53-1	Food processing	[62]
*L. monocytogenes *EGD	Wildtype	^b^
*V. vulnificus *ATCC^T^	Human, clinical	-
*V. parahaemolyticus *ATCC^T^	Human, clinical	-

Susceptibility testing were carried out with a selection of twelve different bacterial strains comprising common laboratory strains and clinical strains derived from food-borne pathogens as well as pathogens responsible for nosocomial infections. ^a ^ESBL-producing clinical samples from Danish patients in 2007; ^b ^This strain was kindly provided by Werner Goebel, University of Würzburg.

### Peptide synthesis and selection

α-Peptide/β-peptoid chimeras consisting of alternating repeats of natural cationic α-amino acids and synthetic lipophilic β-peptoid residues were prepared by solid-phase synthesis as previously described [21,22]. Six chimeras were investigated in this study. The possible differences in sensitivity of different bacterial species were evaluated by testing the analogues 1, 2 and 3, distinguished by different degrees of chirality and type of cationic amino acid. Additionally, the mixed series 4a, 4b and 4c, differing only in the chain length, was used for evaluating the effect of this on antimicrobial activity (Figure 1). Compounds 1, 2 and 3 have been described previously [23,24,29], while the series 4a, 4b and 4c were synthesized using the already established synthesis protocols involving known dimeric building blocks [21,22]. The identity of the primary peptidomimetic sequences 4a, 4b and 4c were confirmed by high-resolution MS (Bruker MicroTOF-Q LC mass spectrometer equipped with an electrospray ionization source): compound 4a, (m/z) [M+4H]^4+ ^obsd. = 339.9727 (calcd. = 339.9719, ΔM 2.3 ppm); compound 4b, (m/z) [M+5H]^5+ ^obsd. = 402.0614 (calcd. = 402.0608, ΔM 1.4 ppm); compound 4c, (m/z) [M+6H]^6+ ^obsd. = 443.2880 (calcd. = 443.2879, ΔM 0.2 ppm). Peptides were solubilized to a stock of 10 mg/mL in sterile MilliQ water and stored at -20°C.

### Determination of Minimum Inhibitory Concentration (MIC) and Minimum Bactericidal Concentration (MBC)

The Minimum Inhibitory Concentration (MIC) of the chimeras was determined against the spectrum of bacteria using the microdilution method according to guidelines of the Clinical and Laboratory Standards Institute (CLSI) [30]. Chimera 1:2 serial dilutions were prepared from 1,024 μg/mL stock solutions to give a final range of 512-0.5 μg/mL in the wells. This corresponds to a final range of 144 to 0.14 μM for the heaviest chimera (i.e. chimera 4c) and of 282 to 0.27 μM for the lightest chimera (i.e. chimera 4a). Colonies grown overnight (i.e. approximately 18 hours) on BHI agar were suspended in 0.9% saline to give a turbidity of 0.13 at OD_546 _(approximately 1 × 10^8 ^CFU/mL), and then diluted in MHB pH 7.4 to a final concentration of 5 × 10^5 ^CFU/mL in each well. Following CLSI guidelines the media for testing of *Listeria monocytogenes *strains were supplemented with 2.5% lysed horse blood. Polypropylene plates (Nunc 442587) were used to minimize peptide binding and incubation time was 18-20 hours at 37°C. MIC was determined in a minimum of two technical replicates as the lowest concentration of the peptide analogue where no visible growth was found. The Minimum Bactericidal Concentration (MBC) was determined by plating 10 μL of the suspension from the first three wells without growth on BHI agar and incubating these for 24 hours at 37°C. MBC was the lowest concentration at which a 99.9% reduction in CFU/mL was observed. Activity is expressed in μmol/L to enable a direct comparison of analogues with different length (= size).

### Killing kinetics of *Staphylococcus aureus *and *Serratia marcescens*

*In vitro *time-kill curves for chimera 1, 2 and 3 were determined against *S. aureus *8325 (MIC μM: chimera 1 5.9; chimera 2 2.8; chimera 3 18.7) and *Serratia marcescens *ATCC 8100 (MIC μM: chimera 1 46.8; chimera 2 45.5; chimera 3 150.0). These two bacterial strains represent organisms susceptible and tolerant to the chimeras, respectively. The bactericidal effect of the three chimeras was tested at MIC in two independent experiments; additionally the effect of chimera 2 was tested at ¼ and 1/2 times MIC. In brief, a suspension prepared from fresh overnight colonies as described above was transferred to 2 mL PBS or cation-adjusted MHB with chimera added (from a 10 × MIC solution) to give a similar bacterial cell density as employed in the MIC determination; the resulting suspension was then incubated at 37°C, 300 rpm. Samples for colony determination were taken at 0, 1, 2, 4, 6 and 8 hours after addition and transferred to a ten-fold dilution row. Colony counts were determined after incubation for 24 hours at 37°C.

### ATP leakage assay

Pore formation as caused by peptide addition was determined by measuring ATP leakage from the bacterial cell using a bioluminescence assay [31]. The assay was used to estimate differences between sub-typical chimeras 1, 2 and 3 on *S. aureus *and *S. marcescens *and to evaluate the effect of chain length of mixed type chimeras 4a, 4b and 4c on *S. aureus*. In brief, bacteria were grown in TSB at 37°C for 24 hours and then re-inoculated in TSB at 37°C for 6-8 hours until an absorbance at 546 nm of 2.5 for *S. aureus *and 2.0 for *S. marcescens *and then harvested (10 min at 2,000 × g). The bacteria were grown to a high absorbance since a high concentration of bacteria was necessary in order to get a measurable response in the ATP leakage assay. Cells were washed once in 50 mM potassium phosphate buffer (pH 7.0) and once in 50 mM HEPES buffer (pH 7.0), before the pellet was resuspended in HEPES buffer to an OD_546 _~ 10, and then stored on ice. Before chimera addition bacteria were pre-incubated with 0.2% (w/v) glucose to energize the cells. In general a chimera dose of 1000 μg/mL (corresponding to 280-552 μM for all chimeras) was used for all assays; however, for determining dose response curves additional doses of 100 (28-55 μM), 250 (71-137 μM) and 500 (140-276 μM) μg/mL were tested, and only the immediate release was noted. Total ATP and extracellular ATP were determined with a luminometer (Pharmacia Biotech Novaspec II Visible Spectrophotometer). Intracellular volumes [32] of *S. aureus *and *S. marcescens *(0.85 μm^3 ^and 1.7 μm^3^, respectively) were subtracted from the total volume before calculating the extracellular ATP concentration; the intracellular ATP concentration could then be calculated from this and the total ATP. ATP leakage kinetics was determined on a bacterial suspension prepared as above. Samples were taken at time 0, 5, 10, 20, 30 and 60 minutes and viable counts determined. Both the ATP leakage assay and killing kinetics performed under the same assay conditions were performed in two independent experiments.

## Results

Based on our previously published work on α-peptide/β-peptoid chimeras [23,24,29] we selected six compounds for the present study. Our main purpose was to examine the influence of the type of cationic amino acid and chain length on antibacterial activity and specificity. Also we aimed at elucidating the mechanism of action against live bacterial cells and determine if this (membrane perturbation) was influenced by the chimera structural characteristics. We measured ATP leakage from chimera-treated cells as an indication of membrane pertubation. Comparing the ATP leakage with time-kill studies allowed us to establish if there was a direct correlation between permeabilization of the membrane and killing of bacterial cells.

### MIC and MBC against clinical and food-borne pathogens

Twelve strains representing seven bacterial species were tested for their susceptibility to the peptide analogues. The analogues exhibited a broad-spectrum activity with no distinct differences between Gram-positive and -negative bacteria (Table 2). Five of the six chimeras had a strong antibacterial effect with MIC values below 5 μM. Important food-borne pathogens were included in the susceptibility assay panel. Thus, three *L. monocytogenes *strains representing both a clinical lineage 1 strain (strain 4446) and a persistent lineage 2 strain from a food-processing plant (strain N53-1) as well as clinical isolates of *V. vulnificus *and *V. parahaemolyticus *were examined.

**Table 2:** Minimum Inhibitory Concentration (μM) of the six α-peptide/β-peptoid chimeras in the present study

	Chimera 1	Chimera 2	Chimera 3	Chimera 4a	Chimera 4b	Chimera 4c
*S. aureus *8325	5.9	2.8	18.7	141.2	23.8	4.5
*K. pneumoniae *ATCC 13883	1.5	2.8	37.5	282.4	23.8	9.0
*S. marcescens *ATCC 8100	46.8	45.5	150.0	> 282.4	190.3	71.8
*E. coli *ATCC 25922	1.5	2.8	9.4	141.2	3.0	2.2
*E. coli *MG1655	1.5	2.8	4.7	141.2	5.9	2.2
*E. coli *AAS-EC-009	1.5	2.8	9.4	141.2	11.9	4.5
*E.coli *AAS-EC-010	1.5	1.4	9.4	141.2	3.0	2.2
*L. monocytogenes *4446	2.9	1.4	1.1	70.6	3.0	1.1
*L. monocytogenes *N53-1	2.9	2.8	1.1	70.6	5.9	1.1
*L. monocytogenes *EGD	1.5	2.8	1.1	70.6	3.0	1.1
*V. vulnificus *ATCC^T^	1.5	1.4	2.3	35.3	3.0	2.2
*V. parahaemolyticus *ATCC^T^	1.5	1.4	2.3	70.6	3.0	1.1

Minimum Inhibitory Concentration of the six peptidomimetics in this study against the spectrum of bacteria expressed in μM. Values were obtained from a minimum of two independent trials. The Minimum Bactericidal Concentration (MBC) was in all assays equal to or a maximum of one two-fold higher than the MIC value indicating a bactericidal mode of action.

The MIC values of chimeras 1, 2 and 3 were similar, indicating that the β-peptoid side chain chirality (i.e. 1 vs. 2) had no effect on antibacterial activity and that the 12-meric homoarginine (hArg) based sequence 2 was likely equalled by the longer 16-meric lysine-containing analogue 3. Generally, low MIC values were found for these three compounds, however, the activity of chimera 3 was slightly lower than for chimera 1 and 2 against some of the bacteria i.e. *S. aureus*, *K. pneumoniae *and *S. marcescens*.

Chimeras 4a, 4b and 4c all have a 1:1 mixture of Lys and hArg residues, but differ in length (8-16 residues), and this had a marked effect on their antibacterial activity. The pattern was the same against all bacterial strains tested. The longest of the three, chimera 4c, was the most active compound with MIC values of 1.1-2.2 μM against the food-borne pathogens *L. monocytogenes *and *Vibro spp*. Chimera 4c was also active against the clinical strains of *E. coli*, *S. aureus *and *K. pneumoniae *with MIC values in the range of 2.2-9.0 μM (Table 2). Chimera 4b, with a length of 12 residues, was less antibacterial with MIC values approximately 2-3 times higher than those of the 16-mer 4c (Table 2). Chimera 4a being only half the length of chimera 4c was the least antibacterial as the MIC values were 15-70 times higher than those of chimera 4c (Table 2). Thus, the relative increase in activity was much larger for elongation with a third repeating unit (i.e. from 8-mer 4a to 12-mer 4b), than the further elongation of 4b with a fourth repeating unit to afford 4c, revealing the minimally required length of an active AMP analogue to be approximately 12 residues.

Two Extended Spectrum Beta-Lactamase (ESBL)-producing *E. coli *clinical isolates (AAS-EC-009 and AAS-EC-010) were included to determine if this antibiotic resistance affected chimera sensitivity. However, the chimeras were as effective against these strains as against non-ESBL strains indicating that resistance mechanisms conferring resistance to conventional antibiotics do not diminish the activity of the present peptidomimetics. Interestingly, *S. marcescens*, which is known to be intrinsically resistant to other antimicrobial peptides, was tolerant to all six chimeras (MICs above 46 μM; Table 2), and it most likely possesses resistance mechanisms that are different from those present in the two multi-resistant *E. coli *strains.

All six chimeras had a Minimum Bactericidal Concentration (MBC) equal to or double the MIC. The high similarity between the MIC and MBC values indicates that the chimeras exhibit a bactericidal mode of action.

### Killing kinetics in two bacteria with different susceptibility

*S. marcescens *was the only bacterial strain tested that was tolerant to the α-peptide/β-peptoid chimeras. The strain is the only one considered intrinsically resistant to the polymyxin group of AMPs, and this could explain its resistance to our peptidomimetics. If so, this would indicate that a very similar resistance mechanism was responsible for the observed decrease in susceptibility. Therefore we performed a comparative mechanistic study that also included *S. aureus *and *E. coli *as susceptible reference strains.

**Figure 2:**
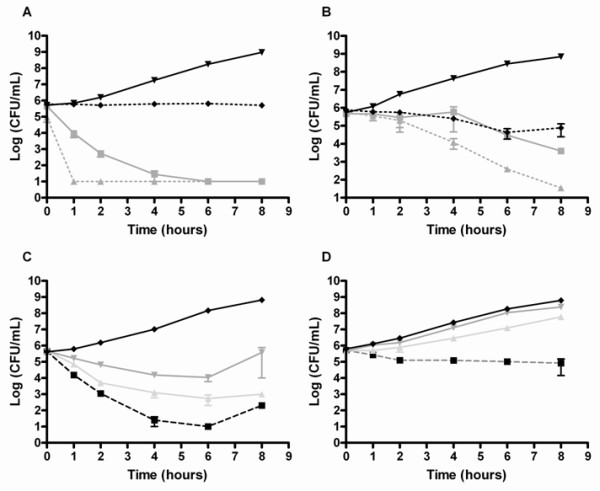
**Killing kinetics of chimera 2 against *S. marcescens *(A+C) and *S. aureus *(B+D) displayed as mean number of viable cells with standard error of the mean (SEM)**. The assays were performed in two independent experiments. Time-kill of the chimera was determined at MIC in MHB (grey solid) and PBS (grey punctuated) and compared to MilliQ-treated control in MHB (black solid) and PBS (black punctuated) for *S. marcescens *(A) and *S. aureus *(B). The effect of chimera concentration on time-kill was determined in MHB at ¼ MIC (dark grey), 1/2 MIC (light grey) and MIC (black punctuated) and compared with MilliQ-treated control (black solid) for *S. marcescens *and (C) and *S. aureus *(D).

We exposed *S. aureus *and *S. marcescens *to peptidomimetics 1, 2 and 3 at three different concentrations in MHB as well as at their MIC concentration in PBS buffer in order to determine whether these chimeras were only active against growing bacterial cells. *S. marcescens *was killed rapidly by chimera 2 (Figure 2A), and the lethal effect was clearly concentration-dependent (Figure 2C). In contrast, *S. aureus *was killed more slowly and with a less pronounced effect of dose (Figure 2B and 2D). Treatment of *S. marcescens *with chimera 2 at its MIC caused a 2 log decrease in the number of viable bacteria within the first hour after which cell numbers declined over the next 5 hours. When the bacteria were treated with the chimera in PBS, the killing occurred very rapidly and no viable cells remained after the first hour. When *S. aureus *was treated with chimera 2 at the MIC in MHB, the number of viable cells did not decrease until after 6 hours, however, when treated in PBS, viable cell numbers decreased with log 2 after 4 hours (Figure 2B). Even though a slightly decreased growth rate was observed for *S. aureus *upon treatment with concentrations below MIC as compared to the control, a concentration close to the MIC value was needed to completely inhibit growth of the culture (Figure 2D). In comparison, as low as ¼ MIC resulted in a reduction in cell number of *S. marcescens *(Figure 2C) revealing a more pronounced concentration-dependent killing for this bacterium.

Since the MIC value found for *S. marcescens *was considerably higher than that seen for *S. aureus*, we performed time-kill on *E. coli*, which exhibited a similar susceptibility in terms of MIC to that of *S. aureus*, to test if the rapid lethal effect against *S. marcescens *was due to the higher concentrations of peptidomimetics (*E. coli *ATCC 25922 MIC μM: chimera 1 1.5; chimera 2 2.8; chimera 3 9.4). However, a rapid killing effect was also found for this bacterial species (data not shown) ruling out that the elevated concentrations solely could be responsible for the high killing rate seen for *S. marcescens*.

### Membrane perturbation effects in two bacteria with different sensitivity

Killing kinetics often reflect the mode of action, and we hypothesized that differences between *S. aureus *and *S. marcescens *regarding their sensitivity and time-kill might be due to different modes of interaction with the peptidomimetics. Therefore, an ATP bioluminescence assay was employed to determine (i) whether cell envelope perturbation was involved in the antibacterial effect, and (ii) if so, whether the organisms differed in the degree of ATP leakage.

**Figure 3:**
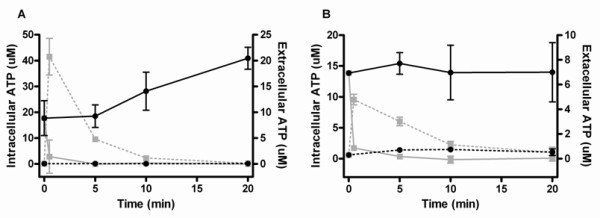
**Chimera-induced ATP leakage in *S. aureus *(A) and *S. marcescens *(B) after treatment with 1000 μg/mL chimera**. The assays were performed in two independent experiments. Mean (SEM) intracellular (IC, solid line) and extracellular (EC, punctuated line) ATP concentration for *S. aureus *cells (figure A, grey lines) and *S. marcescens *cells (figure B, grey lines) treated with chimera 1 compared to MilliQ-treated control (black lines).

Chimera 1, 2 and 3 caused leakage of ATP from both *S. aureus *and *S. marcescens*, but all three peptidomimetics gave rise to an ATP leakage from *S. aureus *that was substantially larger than that from *S. marcescens *(see Figure 3 for results with chimera 1). The intracellular ATP concentration rapidly approached zero for both bacteria within the first few minutes, whereas the extracellular ATP concentration increased more rapidly during the first minutes for *S. aureus *(~20 μM) than for *S. marcescens *(~5 μM). To examine if this could be due to the fact that the two bacteria were treated with the same dose despite their very different MIC values, we determined their dose response curves. For both bacteria a minimum chimera dose of 500 μg/mL (i.e. 145-180 μM) was needed to obtain the maximum immediate response (data not shown) ruling out that the rapid release of ATP from *S. aureus *seen in Figure 3A is due to a higher concentration/MIC ratio than employed for *S. marcescens*.

To investigate if the degree of ATP leakage from the bacterial cell corresponded to the simultaneous decrease in the number of viable cells (i.e. if *S. marcescens *cells on the basis of their elevated MIC were in fact able to survive even after a moderate ATP leakage) we determined time-kill under *exactly *the same conditions as the ATP bioluminescence assay had been performed. Irrespective of which of the three chimeras that were used, both bacteria were reduced 2-3 log from an initial value of log ~9.5 per mL within the first 20 minutes before the ATP leakage tailored off and no further decrease in viable count was seen for up to 60 minutes (not shown). This indicates that the degree of ATP leakage from the two bacteria (i.e. the concentration of the extracellular ATP) does *not *reflect differences in viability. No reduction in the number of viable bacteria was seen for the control (not shown), and the intracellular concentration of ATP did not change (Figure 3A and 3B).

Although there was no systematic difference in the MIC values between Gram-positive and -negative bacteria, we speculated that the Gram-negative outer membrane could act as a barrier to the penetration of AMPs, since polymyxin B resistance in *S. marcescens *has been linked to induced changes in the amount and composition of lipopolysaccharide (LPS) in the outer membrane [33]. Moreover, similar resistance-conferring membrane alterations have also been seen for other bacteria in response to polymyxin B treatment [34-36]. Accordingly, we studied how a membrane-destabilizing pre-treatment of *S. marcescens*, *E. coli *and *S. aureus *with the divalent metal cation-chelating agent EDTA would affect the killing caused by chimera 1. In these experiments we used a non-lethal 0.5 mM concentration of EDTA together with the non-lethal 1.5 μM concentration of the tested AMP analogue. A slight reduction in the number of viable cells corresponding to 0.5 log was seen for *S. aureus *when treated with chimera 1 alone while *E. coli *and *S. marcescens *were reduced with 1.5 log (data not shown). No discernable difference in the number of viable cells remaining was observed between *S. aureus *treated successively with EDTA and peptidomimetic and *S. aureus *treated only with the peptidomimetic. In contrast, cell numbers of both *S. marcescens *and *E. coli *were reduced with 4-5 log from an initial value of log ~5.5 within the first 4 hours (not shown) upon treatment with a sub-lethal EDTA concentration together with the chimera. This indicates that the intact outer membrane indeed appears to act as a protective barrier against the antibacterial chimeras.

### The effect of chimera chain length on membrane perturbation activity

Peptidomimetics 4a, 4b and 4c consist of the same repeating unit of four residues (Figure 1; n = 2, 3 and 4, respectively), and thus differ only in length. The MIC values increased dramatically when going from 8-mer (4a) to 12-mer (4b) while further elongation to 16-mer (4c) only led to a slight enhancement in potency (Table 2). Hence, we were intrigued to establish whether mechanistic differences could explain this strong correlation.

**Figure 4:**
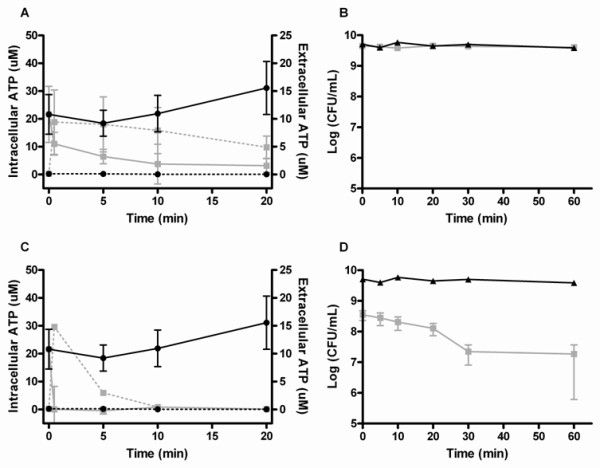
**The effect of chimera chain length on ATP release from *S. aureus *after treatment with 1000 μg/mL chimera and the corresponding change in the number of viable cells after treatment with chimera 4a (A+B) or chimera 4c (C+D)**. The assays were performed in two independent experiments. Mean (SEM) intracellular (IC, solid line) and extracellular (EC, punctuated line) ATP concentration for cells treated with chimera 4a (figure A, grey lines) or 4c (figure C, grey lines) compared to MilliQ-treated control (black lines). Mean (SEM) number of viable cells after addition of chimera 4a (figure B, grey line) or 4c (figure D, grey line) compared to MilliQ-treated control (black line).

We determined ATP leakage from *S. aureus *when treated with chimeras 4a, 4b and 4c to evaluate the effect of chain length on the extent of pore formation or membrane disintegration caused by the chimeras. Peptidomimetic-induced ATP leakage was markedly different for *S. aureus *treated with chimera 4a (Figure 4A) as compared to *S. aureus *treated with chimera 4c (Figure 4C). The immediate ATP release was approximately 15 μM for both peptidomimetics; however, the intracellular ATP concentration remained at approx. 5 μM, when the bacterial cells were treated with the shorter analogue 4a, whereas cells treated with chimera 4c were immediately depleted of intracellular ATP. Since the leakage was continuous it seemed that the cells were able to maintain the ATP production. *S. aureus *cells treated with the intermediate length 12-meric chimera 4b had the same leakage pattern as induced by chimera 4a. Dose-response profiles were also determined (as already described in the previous section), and despite differences in MIC values between chimeras 4a and 4c, both reached the immediate maximum ATP release at 500 μg/mL (i.e. 276 μM and 140 μM, respectively). Likewise, the observed ATP release was similar immediately upon treatment with either chimera 4a or 4c, and again cells treated with chimera 4a were able to maintain a low intracellular level of ATP.

The fact that some ATP remained in the cell after treatment with chimera 4a could point to an incomplete disruption of the bacterial cell membrane as compared to bacterial cells treated with chimera 4c. To determine if an intracellular ATP concentration of 5 μM had a physiological effect and would allow the bacterial cells to survive, time-kill was again performed under *exactly *the same conditions as used in the ATP assay to allow comparison of ATP leakage with killing kinetics. After treatment with chimera 4c, cell numbers were reduced with 2 log within the first 20 minutes (Figure 4D), however, after treatment with chimera 4a (Figure 4B) or chimera 4b (not shown) no killing was observed. The pool of intracellular ATP in the peptidomimetic-treated bacterial cells can therefore, as opposed to the amount of leaked ATP, be considered as indicative for the number of viable cells remaining.

## Discussion

The aim of this study was to determine the mechanism of action for a series of peptidomimetics, and specifically we set out to probe the importance of amino acid composition and chain length for antibacterial activity. We included a strain intrinsically resistant to AMPs, and addressed whether killing kinetics and AMP mechanism of action in viable bacteria could provide a mechanistic explanation for the much lower susceptibility of *S. marcescens *as compared to the more sensitive bacteria.

We examined the effect of having exclusively lysine or homoarginine cationic residues as well as of substituting the chiral β-peptoids with achiral counterparts as represented by the α-peptide/β-peptoid chimeras 1, 2 and 3 (Table 2). All three peptidomimetics had MIC values of 1-3 μM against most bacterial strains, which compared to many natural AMPs is a high activity [14,19,37-39]. Noticeably, a considerably lower activity against *S. aureus *and *K. pneumoniae *was observed for the lysine-containing chimera 3 (6-13 fold) as compared to the homoarginine-based chimera 2, while only a slightly lower activity of chimera 3 (2-7 fold) was seen compared to chimera 2 when tested against *E.coli*. The reduced chirality in chimera 1 did not give rise to any significant loss of activity as compared to chimera 2. In a preliminary antimicrobial characterization these peptidomimetics were tested against four common bacteria and a fungus [23], whereas the present study also included important food-borne pathogens *L. monocytogenes*, *V. vulnificus *and *V. parahaemolyticus *against which the chimeras also were active (Table 2).

Additionally we investigated the effect of chain length on activity by studying a series of three peptidomimetics (i.e. chimera 4a, 4b and 4c based on the same repeating unit of four residues), which indicated that the minimally required length for an active peptidomimetic is around 12 residues (Table 2). It has previously been reported that 14 amino acids is the minimal sequence required for an active antimicrobial peptide [25], however, this and other studies focused on the effect of length on helicity which implies structural restrictions in the design to enable it to span the lipid bilayer [26,40]. Also, it is clearly established that the low activity earlier reported for the shorter homologues of chimera 3 (e.g. the 12-mer exhibited almost no activity [23]) may be compensated for by a longer sequence. Chimera 4c corresponds to the analogue where half of the lysines in chimera 3 are replaced by homoarginines, and similarly chimera 4b may be considered an analogue derived from chimera 2 by exchanging half of the homoarginines with lysines. Comparison of the activities found for these two pairs indicates that a high content of homoarginines generally induces a somewhat higher potency; especially, the activity against *S. aureus *and *K. pneumoniae *is clearly promoted by a prevalence of guaninido-functionalized residues.

A high activity was also found against two isolates of ESBL-producing *E. coli *(AAS-EC-09 and AAS-EC-010) indicating that resistance towards conventional antibiotics do not affect the sensitivity towards these peptidomimetics, further supporting a different mode of action. Many AMPs exhibit a cell envelope-perturbing effect [41-43], and hence their target is different from traditional antibiotics of which many act by inhibiting cell wall synthesis or on intracellular targets [44-46]. Notably, *S. marcescens *was the only bacterial strain that proved tolerant to the peptidomimetics, and thus must harbour specific resistance mechanisms involving induction of changes in the cell envelope.

Time-kill experiments showed that *S. marcescens *was killed more rapidly than the susceptible strain of *S. aureus *when treated with chimera 1, 2 or 3 at concentrations close to their MIC values (Figure 2). Polymyxin B and other cationic AMPs may at high doses in themselves act like chelating agents allowing them to penetrate the outer membrane [47,48], however, a noticeable effect was also seen against *S. marcescens *at concentrations lower that the MIC value (Figure 2C). Rapid killing was also demonstrated for *E. coli *exposed to the peptidomimetics, indicating that this could be a phenomenon associated with Gram-negative bacteria. Shorter exposure times caused a significant killing of Gram-negative bacteria when treated with some α-helical AMPs that act by permeabilization of the membrane [37]. Another explanation for the observed differences in the rate of killing could be that either the degree or mode of membrane disruption differs among bacteria i.e. the chimeras may exert their effect by a combination of several mechanisms. The fact that cell membranes of different bacteria differs in lipid composition [49] could influence the interaction between phospholipids and AMPs. However, there is no unequivocal evidence demonstrating that an AMP may exhibit different pore-forming properties in different bacteria, as the proposed co-existence of several disruption modes in fact still is a topic of debate [50,51].

Many AMPs exert their antibacterial effect by interactions with the bacterial cell membrane [38,41,52] involving pore formation or membrane disintegration that in turn causes leakage of the cell contents, which ultimately leads to cell death. Nevertheless, there is a growing amount of indirect evidence that the mechanisms of some very potent AMPs in fact involves an initial period of intracellular accumulation prior to the actual bacterial killing indicating that they act on intracellular targets [38,53,54]. To further investigate the effect of the present peptidomimetics on the cell membrane in *S. marcescens *and *S. aureus *and to determine how structural features of these peptidomimetics might affect the potential membrane-related mode of action we examined their ability to cause leakage of intracellular compounds e.g. ATP. A considerable body of data on the leakage of intracellular compounds has already been obtained by using model membranes thus confirming that many membrane-active peptides indeed exert a permeabilizing effect [24-26,28]. These studies have, however, not demonstrated whether there is a direct kinetic relationship between cell membrane damage and loss of viability, and for this reason we combined leakage assays with a time-kill experiment under exactly the same conditions.

Treatment of both *S. marcescens *and *S. aureus *with peptidomimetics 1, 2 and 3 caused leakage of ATP from the bacterial cells with a similar simultaneous reduction in the number of viable cells, and therefore we conclude that even though *S. marcescens *is tolerant to the peptidomimetics their mode of action against this bacterium is similar to that of *S. aureus*. Earlier, chimera 3 was investigated for its ability to induce calcein leakage in unilamellar liposomes mimicking human cell membranes with a positive response [24], but based on the consistent results in the present work all three peptidomimetics are likely to permeabilize both model and bacterial membranes. Leakage of intracellular compounds has been determined to be the mode of action for many AMPs [55-57], but here we have established this mode of action for a series of peptidomimetics. We conclude that variation of the type of cationic amino acid (i.e. lysine versus homoarginine) did not have an effect on the mode of action in viable bacteria.

Since *S. marcescens *was tolerant to all peptidomimetics tested, their mode of action must therefore involve a target that is ultimately changed by resistance mechanisms in this species. It is well-known that *S. marcescens *is tolerant to the polymyxin group of antimicrobials, and the main hypothesis is that this is due to inherent changes in the composition of the LPS of the Gram-negative outer membrane that acts as a barrier [33]. We demonstrated that the outer membrane also seems to play an important role in the tolerance of *S. marcescens *towards our chimeras as a combined treatment including the chelating agent EDTA resulted in a reduction in the number of viable cells comparable to that seen for a more susceptible Gram-negative strain of *E. coli *treated similarly (not shown). This indicated that the innate differences in susceptibility between the two Gram-negative species could be completely eliminated after destabilization of the outer membrane.

When designing new antimicrobial peptides it is generally accepted that a minimum length is required in order for the peptide to span or transverse the cell membrane. However, the majority of studies have focused on optimizing the length of AMPs assuming it to adopt a helical conformation [25,26,40]. By contrast, due to their design with alternating hydrophobic and cationic residues our peptidomimetics are not expected to adopt an amphipathic helical active confirmation, but rather an extended conformation with some degree of secondary structure as indicated by analysis of their CD spectra [22,23]. Recently, it has been shown that neither global amphipathicity nor regular secondary structure may be required for short peptides to effectively interact with bacterial membranes [19,58], but the optimal length of such peptides has not been rationalized by mechanistic experiments. Only oligomers with a chain length above 12 residues, i.e. the 16-meric peptidomimetic 4c were able to cause such a substantial leakage of ATP that the number of viable cells were reduced (Figure 4C and 4D). We attribute this to the inability of chimeras 4a and 4b to produce a critical degree of membrane disruption thus leaving a sufficient level of intracellular ATP for the cells to survive (Figure 4A and 4B for chimera 4a).

This is to our knowledge the first time that the effect of chain length has been investigated on the membrane-perturbing activity of peptidomimetics without a dominant secondary structure. Also, we believe that our study is the first that directly, in a kinetic fashion, correlate membrane permeabilization with actual killing kinetics.

Previously, the interaction of α-peptide/β-peptides chimeras with liposomal model membranes and murine fibroblast was described [24]. Most recently, we investigated their cytotoxicity and haemolytic activity towards human HeLa cells and erythrocytes, respectively [23]. Besides confirming that members of this subclass of peptidomimetics exhibit a broad antimicrobial activity that includes resistant strains and food-borne pathogens, the purpose of the present study was to undertake a more detailed investigation of their mode of action. The present contribution describes their interaction with viable bacterial cells, and we found that these antimicrobial peptidomimetics have a mode of action involving the cell membrane. The observed membrane disruption depends strongly on chain length, and it may be impeded if the outer membrane in a Gram-negative bacterium possesses an innate altered composition.

## Conclusion

Several α-peptide/β-peptoid chimeras were bactericidal against important food-borne and clinical pathogens with MIC values in the range of 1-5 μM. We examined the effect of changing the ratio between amino- and guanidino-functionalized cationic residues as well as the influence of chain length on both antibacterial activity and ATP leakage. Although, minor differences in the antimicrobial profile of the chimeras may be ascribed to the degree of chirality and/or type of cationic amino acids, by far the most pronounced impact stems from the chain length. Only one bacterial species, *S. marcescens*, was tolerant to the peptidomimetics most likely due to the composition of its outer membrane; however, the ATP leakage was as pronounced as seen for more sensitive bacteria. We conclude that these synthetic antimicrobial peptidomimetics exert their effect through permeabilization of the cell membrane, and that this corresponds to a simultaneous reduction in the number of viable bacteria with the pool of intracellular ATP being indicative of viability. This is the first time that a relationship is established between permeabilization and killing within a peptidomimetics library.

## Competing interests

The authors declare that they have no competing interests.

## Authors' contributions

LHK planned and carried out all experiments and drafted the manuscript. HF designed the peptidomimetics and participated in the revision of the manuscript. KMK synthesized the peptidomimetics. LG helped in the design of the experiments and the drafting of the manuscript. All authors have seen and approved the final manuscript.
